# *Sahaj Samadhi* meditation vs a Health Enhancement Program in improving late-life depression severity and executive function: study protocol for a two-site, randomized controlled trial

**DOI:** 10.1186/s13063-019-3682-z

**Published:** 2019-10-24

**Authors:** Stephen Benjamin Peckham, Emily Ionson, Marouane Nassim, Kevin Ojha, Lena Palaniyappan, Joe Gati, Jean Thebérge, Andrea Lazosky, Mark Speechley, Imants Barušs, Soham Rej, Akshya Vasudev

**Affiliations:** 1 Geriatric Mood Disorders Laboratory, Lawson Health Research Institute, London Health Sciences Centre, London, ON Canada; 2 McGill Meditation and Mind-Body Medicine Research Clinic (MMMM-RC), Jewish General Hospital, Montréal, QC Canada; 3 Geri-PARTy Research Group, Department of Psychiatry, Jewish General Hospital, Montréal, QC Canada; 4 Western University, London, ON Canada; 5 Department of Psychiatry, Schulich School of Medicine and Dentistry, Western University, London, ON Canada; 6 Robarts Research Institute, Western University, London, ON Canada; 7 Imaging Division, Lawson Health Research Institute, London, ON Canada; 8 Department of Medical Biophysics and Medical Imaging, Western University, London, ON Canada; 9 Department of Diagnostic Imaging, St. Joseph’s Health Care, London, ON Canada; 10 London Health Sciences Centre, #A2-607, Victoria Hospital, LHSC, 800 Commissioners Road East, N6A 5W9 London, ON Canada; 11 Department of Epidemiology and Biostatistics, Schulich School of Medicine and Dentistry, Western University, London, ON Canada; 12 Schulich Interfaculty Program in Public Health, Schulich School of Medicine and Dentistry, Western University, London, ON Canada; 13 Department of Psychology, King’s University College, Western University, London, ON Canada

**Keywords:** Late-life depression, Treatment-resistant late-life depression, Executive functioning, *Sahaj Samadhi* meditation, Health Enhancement Program, Randomized controlled trial

## Abstract

**Background:**

Recent estimates suggest an 11% prevalence of current late-life depression (LLD) and a lifetime prevalence of 16–20%. LLD leads to cognitive disturbance as well as a nearly two to three times increased risk of dementia. We conducted a recent randomized controlled trial (RCT) which demonstrated that *Sahaj Samadhi* meditation (SSM), an easy-to-implement, meditation-based augmentation strategy, led to higher rates of symptom remission when compared to treatment as usual (40.0 vs 16.3%; odds ratio, 3.36; 95% CI 1.06–10.64; *p* = 0.040). Here we present a protocol describing a two-site, blinded, RCT, comparing an SSM arm to an active-control arm – a Health Enhancement Program (HEP) intervention – in their ability to reduce depressive symptoms and improve executive functioning, among several other exploratory outcomes.

**Methods/design:**

One hundred and ninety-two (*n* = 192) participants with LLD will be recruited at two sites (London, ON, Canada, and Montreal, QC, Canada). Participants will undergo stratified randomization with regards to site and the presence of treatment-resistant-LLD (TR-LLD) or not, to either SSM or HEP. We will assess change in (1) depression severity using the Hamilton Depression Rating Scale (HAM-D), (2) executive functioning, and (3) other exploratory physiological and mood-based measures, at baseline (0 weeks), post intervention (12 weeks), and 26 weeks after baseline. Raters, clinicians, and care providers will be blinded to group allocation while participants will be blinded to the study hypotheses.

**Discussion:**

This study should more definitively assess whether SSM can be used as an augmentation strategy in routine clinical care for patients suffering from LLD and TR-LLD. If the effects of SSM are significantly better than HEP, it will offer support for the routine use of this intervention to manage LLD/TR-LLD and comorbid declines in executive dysfunction. The results of this study could also inform whether SSM can improve/prevent cognitive decline in LLD.

**Trial registration:**

ClinicalTrials.gov, ID: NCT03564041. Registered on 20 June 2018.

## Background

### Late-life depression

The older adult population is growing worldwide with those aged 60 years and older to comprise over 22% of the global demography by 2050 [1]. In some regions of the world, such as North America, this shift is predicted to occur within the next 10 years [2–4]. An estimated 15% of individuals within this age group are currently living with a mental illness [5]. Late-life depression (LLD), specifically, has been found to have a current prevalence of 11% [6] and a lifetime prevalence of 16–20% [7, 8]. Direct healthcare costs incurred through treatment of LLD patients are approximately 33% higher than for their non-depressed counterparts [9] and have been reported to be as high as CAD$5 billion per year in Canada, alone [10]. Indirect costs (e.g., informal caregiving; suicide) have been reported to be as high as CAD$1.8 billion [11]. These considerable costs could be reduced by the successful treatment of LLD and its treatment-resistant variant (TR-LLD).

The standard treatment for LLD is the use of antidepressants, such as selective serotonin-reuptake inhibitors (SSRIs), and/or psychotherapy. These current treatments, however, have significant limitations. Patients are frequently not able to tolerate adequate doses and, as such, there is often poor compliance with pharmaceutical-based treatment plans. In addition, the monoamine theory of depression does not seem to address other psychosocial circumstances which have been implicated in the precipitation and maintenance of depression [7]. Given the above, it is no surprise that treatment resistance (TR) is prevalent in up to 60% of patients with LLD [12]. Also, it has also been shown that as many as 30% of patients with LLD discontinue their antidepressants within 6 months of initiation due to drug-related adverse effects [12].

Alternatives to pharmaceutical management of LLD include various psychotherapy interventions. Such evidence-based interventions include problem-solving therapy, interpersonal therapy, and cognitive behavioral therapy [12, 13]. Despite the efficacy of these therapies there is often a lack of trained professionals, inability to access this care, or, in patients where therapy is available, a wait time of up to 18 months [14]. The prevalence of LLD, associated costs, lack of response to pharmacotherapies, and difficulty accessing conventional psychotherapies and pharmacotherapy consultations raises the need to find novel, cost-effective, and scalable interventions for this important mental health disorder.

### Depression, dementia, and other related risks/illnesses

A depressive episode has been found to nearly double a person’s chance of developing dementia as shown by previous meta-analyses [15, 16]. Studies also have demonstrated that 30–50% of LLD patients show impairments in cognitive functioning [17]. Additionally, patients with LLD report impairments in memory, attention, information processing, and executive functioning [16, 18, 19]. Executive functioning, in particular, has been found to be directly associated with LLD [18]. This executive dysfunction persists and significantly interferes with treatment responsiveness in LLD and has been implicated as one of the most important markers for conversion to dementia [20]. In the United States alone, the healthcare costs associated with managing dementia have been calculated to be as high as USD$215 billion per year [21]. Even though there are effective care models established for dementia, its irreversible nature highlights the importance of investigating the preventive potential of various non-pharmacological interventions such as exercise- and meditation-based therapies.

LLD and TR-LLD are also associated with a multitude of health comorbidities. These include: stress [22], anxiety, suicidal ideation [23], lower quality of life, increased medical comorbidity, and higher mortality rates compared to age-matched controls [14]. Attempts have been made to understand the neurobiology of LLD and TR-LLD and, so far, a host of brain and other systems have been implicated including cortisol dysregulation [24], cardiovascular autonomic impairment [25], gait disturbances [26–28], increased presence of inflammatory markers [20, 29, 30], sleep disturbances [31, 32], as well as structural and functional brain changes [33–35]. It is hence important to assess changes, if any, in these pathophysiological pathways. It is possible that any new treatment strategy for LLD and/or TR-LLD could lead to reversal of these changes.

### *Sahaj Samadhi* meditation

Recent research has shown benefits of some mind-body (e.g., yoga, *tai chi*, mindfulness) interventions in reducing depressive symptoms across the age span [36–40]. *Sahaj Samadhi* meditation (SSM) is a less-investigated meditation intervention which differs from others as it lets the practitioner attain a deep state of relaxation aided by the use of a personalized mantra, which is to be used only when needed, during the meditation practice. It does not require focus on one’s thoughts or environment, on mindfulness or on frequently repeating a mantra as in focus-based meditation techniques.

The authors conducted a recent randomized controlled trial (RCT) on SSM in LLD compared to a treatment-as-usual (TAU) arm. It was observed that those in the SSM arm had a significantly greater decrease in depressive symptoms compared to the TAU arm (mean point-change difference − 2.66; 95% CI − 5.05 to − 0.26; *p* = 0.030), with 40% of SSM participants entering remission compared to 16.3% in the TAU arm (odds ratio of 3.36; 95% CI 1.06 to 10.64; *p* = 0.040) [37]. There were no reported side effects associated with SSM. Additionally, participants’ qualitative feedback included remarks stating that SSM is easily learned and can be practiced as part of a daily routine. The observed practical benefits of SSM included relative affordability, the ability to be taught in a group setting, and easy transferal to community/clinical settings [37, 39]. SSM may additionally ameliorate the cognitive impairments associated with LLD and TR-LLD as other transcendental meditation techniques – practiced twice a day, 2 days per week, for 12 weeks (as in the present study) – have shown improvements in this domain [41]. Furthermore, there is some pilot data to suggest that the category of meditation to which SSM belongs, namely automatic self-transcendental meditation (ASTM), positively affects immune responses to stress [42].

For the purpose of further exploration of the beneficial effects of SSM we plan to conduct a study with the primary hypothesis that SSM, compared to an active control, will lead to a significant improvement of depressive symptoms in participants with LLD at 12 weeks and that this effect will persist at 26 weeks. We will also examine a secondary hypothesis that SSM leads to an improvement in other variables including executive function. Exploratory outcomes will be changes in anxiety, quality of life, gait, and central as well as peripheral immune function.

## Methods/Design

### Aims

The objective of this study is to compare the effects of *Sahaj Samadhi* meditation (SSM) on related psychological and biological outcomes against an active control, the Health Enhancement Program (HEP).

Primary outcome: the primary outcome measure will be change in depression scores in the SSM arm compared to HEP at 12 weeks. We will also assess the percentage of SSM participants who will meet the clinical criteria for remission compared to HEP at 12 weeks. Lastly, we will assess whether participants in the SSM arm will continue to have reduced depression scores at the 26-week follow-up.

Secondary outcome: the secondary outcome measure will be a neuropsychological battery for assessing executive function with an emphasis on planning and organization ability, at the primary endpoint of 12 weeks.

Exploratory outcomes: for a complete list of such outcomes and their associated hypotheses, please refer to Table 1.

**Table 1:** Objectives and hypotheses

Objective		Hypothesis
Primary		
1.	Using a 12-week RCT, assess whether SSM improves depressive symptoms in LLD compared to an active-control condition; HEP	(a) In participants with LLD, SSM participants, compared to HEP controls, will have a greater reduction in depressive symptoms from baseline to 12-week follow-up, as measured by the Hamilton Rating Scale for Depression (HRSD-17) (b) Compared to HEP, a higher percentage of SSM LLD participants will meet the clinical criteria for remission, as defined by HRSD-17 scores < 8 at the 12-week follow-up
Secondary		
2.	To assess the effects of SSM on a specific executive function, i.e., organization ability in LLD	In participants with LLD, compared to HEP, SSM will be associated with better organization ability, at 12-week follow-up
Exploratory		
3.	To assess the effects of SSM on other executive function domains in LLD	In participants with LLD, compared to HEP, SSM will be associated with better executive functions, at 12-week follow-up
4.	To assess the effects of SSM on global cognition in LLD	In participants with LLD, compared to HEP, SSM will be associated with better global cognition, at 12-week follow-up
5.	Using magnetic resonance spectroscopy (MRS), assess the effects of SSM on functional connectivity measured by rs-fMRI particularly within the area implicated on the default mode network, volume increase in the bilateral hippocampi and posterior cingulate cortices and levels of glutathione (GSH)	Compared to HEP, SSM will show an increase in functional connectivity especially within the area implicated on the default mode network and also a volume increase in the bilateral hippocampi and posterior cingulate cortices and an increase in levels of GSH in key mood-regulating brain areas (ventro-medial prefrontal cortex)
6.	To investigate whether SSM intervention is associated with sustained improvements in participants’ views of disability (WHODAS 2.0), sleep (Athens Insomnia Scale), quality of life (Euro-QOL), anxiety (Generalized Anxiety Disorder GAD-7, and overall psychological well-being (Ryff’s Scales of Psychological Well-Being; SPWB 9 items) at 12-week follow-up compared to HEP	SSM will be superior to HEP at 12 weeks on all self-rated measures of disability, sleep, quality of life, anxiety, and psychological well-being
7.	To investigate the effects of SSM and HEP on mood and cognition at 26-week follow-up	SSM will continue to be superior to HEP at 26-week follow-up with regard to HRSD-17 scores and executive functioning
8.	To identify and quantify the extent of gait impairment in participants with LLD and explore the effects of SSM intervention on gait impairments at 12 weeks	SSM will be superior to HEP in improving gait measures, including stride length, gait velocity and fear of falling and risk of falling as measured by the Falls Efficacy Scale – International (FES-I) at 12-week follow-up
9.	To explore whether blood inflammation markers, circadian rhythm, and sleep quality (Acti-watch), predict treatment response with SSM	Depression and anxiety symptoms will be associated with levels of inflammatory markers. Levels of inflammatory markers will be decreased, as well as circadian rhythm and sleep quality (Acti-watch) will be improved, in the SSM participants at 12-week follow-up compared to HEP, which in turn will be associated with greater reductions in depression/anxiety scores
10.	To assess participant experience after participating in the interventions (SSM or HEP)	Using the semi-structured McGill Illness Narrative Interview, elucidate what is the participant experience participating in either SSM or HEP?
11.	To assess the extent to which participanting in SSM or HEP is associated with any change in mindfulness scores using the Five Facet Mindfulness Questionnaire (FFMQ)	Compared to HEP, SSM is associated with greater increases in FFMQ mindfulness scores. This in turn is associated with greater improvements in HRSD-17 depression scores (see main outcome section)
12.	To explore whether there are any changes in level of consciousness, at week 12, participants will fill out the self-report Phenomenology of Consciousness Inventory (PCI) after their meditation session for the SSM group or analogous activities for the HEP group	Participants practicing SSM will be in an altered state of consciousness compared to participants engaged in HEP

*HEP* Health Enhancement Program, *LLD* late-life depression*, rs-fMRI* resting state  functional magnetic resonance imaging, *RCT* randomized controlled trial, *SSM Sahaj Samadhi* meditation

### Design

The study is a two-site (London, Ontario (ON), Canada, and Montreal, Quebec (QC), Canada), 12-week, RCT in which raters, clinicians, and care providers will be blinded to participant group allocation and participants will be blinded to study hypotheses. Participants will attend three assessment sessions (baseline/0 weeks, 12 weeks, and 26 weeks) in a research-hospital setting.

**Fig. 1:**
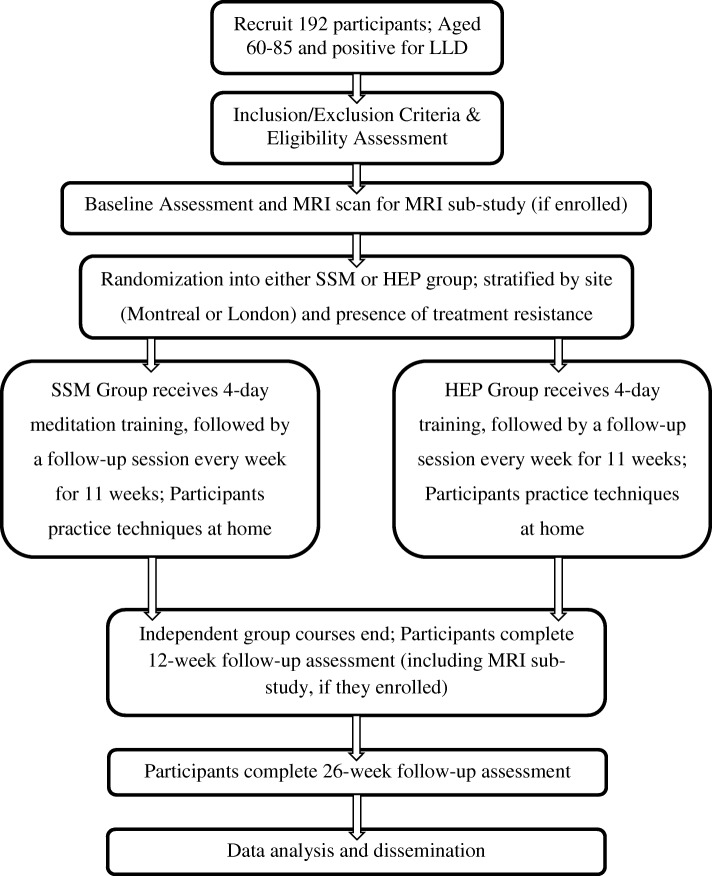
Participant flow chart

Participants will attend either SSM or HEP for four consecutive days of training for 2 h per day. Following the 4 days of initial training, participants will attend 1-h weekly follow-up sessions of HEP or SSM for 11 subsequent weeks. See Fig. 1 for an outline of the study assessments and the intervention schedule. A subset of participants will be recruited in an exploratory sub-study examining neurobiological brain changes associated with depression and any changes in depressive symptoms, with magnetic resonance image (MRI) scanning to take place at the 0-week and 12-week time points. Patients will also be offered the opportunity to participate in sub-studies designed to assess the effects of SSM versus HEP on (1) gait as measured by a standardized gait mat, (2) peripheral inflammatory markers, (3) sleep as measured by a proprietary Actigraph, and (4) change in level of mindfulness, awareness as well as shifts in level of consciousness.

### Recruitment

Potential participants who meet the inclusion/exclusion criteria will be referred by their family physician or any specialists involved in their care. Participants may also hear about the study through strategically placed public advertisements at local community centers, clinics, hospital units, and/or libraries. A member of the research team will initiate contact and provide full information of the study – as a letter of information (LOI) and consent form in London, ON, as well as in Montreal, QC.

We will provide a minimum time interval of 24 h to all potential participants to review the relevant study information. Afterwards, a member of the research staff will contact them to schedule an assessment for eligibility and a baseline data assessment.

Recruitment is expected to occur over a 26- to 36-month period at a rate of two participants per week. The SSM or HEP group interventions will be run only upon enrollment of a minimum sample of eight and a maximum allowable sample of 20 participants. Randomization of each group will be block stratified with a random block size allocation, by study location (London or Montreal) as well as the presence or not of TR (defined below).

### Participants

Research participants will be 192 men and women between 60 and 85 years of age who meet the criteria for a mild or moderate major depressive episode according to a structured clinical interview using the Mini International Neuropsychiatric Interview (MINI) [43]. This will be confirmed by a score of between 10 and 22 on the Hamilton Rating Scale for Depression (HRSD-17) [44, 45].

### Eligibility assessment

Potential participants will be screened as per eligibility criteria in academic hospital settings: Parkwood Mental Health Institute in London, ON, Canada, by the Geriatric Mood Disorders Laboratory; and McGill University (Douglas Hospital, Jewish General Hospital, McGill University Health Care, St. Mary’s sites) in Montreal, QC, Canada, by the GeriPARTy group. Participants who meet the inclusion criteria and who are recruited into the study will be 60–85 years old, present with a mild to moderate major depressive episode, be willing and able to attend four training sessions of the interventions as well as 75% of follow-up sessions, have sufficient language capacity (English in London, ON; English or French in Montreal, QC), have sufficient hearing to listen to instructions, be able to sit for 20–25 min without discomfort, and be willing to remain on the same antidepressants, including dosage, for the first 12 weeks of the study. Treatment resistance will be determined by the Antidepressant Treatment History Form (ATHF). TR status will be assigned to participants who are currently prescribed, or have been prescribed, in the current episode an adequate dose and duration of two different medications listed on the ATHF. An “adequate” dose will be deemed to be a rating of “3” or higher on any ATHF item [46–48].

Participants will be excluded from the study if they are unable to provide informed consent, have a severe personality disorder that would interfere with group functioning, have an IQ below 70 (as estimated by a psychological assessment), have clinically significant sensory impairment, or are currently practicing another mind-body intervention on a regular basis. Participants will also be excluded if they have clinical presence of dementia or another serious mental illness (e.g., bipolar I/II), have active or had recent substance abuse or dependence, and/or have an elevated suicide risk as assessed by the Mini International Neuropsychiatric Interview (MINI) [43].

## Outcomes

A screening assessment will be conducted to determine a participant’s eligibility for the study. Following completion of the informed consent process, baseline assessments will be completed. Demographics collected at baseline include age, biological sex, and self-identified gender, ethnicity, height, weight, along with hip and waist circumference, as well as housing, marital, and employment status. A medication record will be collected as per participant report along with reconciliation from pharmacy records.

The following scales will be administered at the baseline assessments (a complete list of the assessments to be completed at different time points can be found in Table 2).

**Table 2:** Primary and secondary outcome measures for all participants; weeks 0–26

Evaluations	Enrollment	Randomization and interventions’ start	Follow-ups	
	Week 0	Week 1	Week 12	Week 26
Primary outcome: depression				
HRSD-17	✓		✓	✓
Secondary outcome: executive function				
WAIS-IV–Digit Span	✓		✓	✓
D-KEFS Verbal Fluency Test	✓		✓	✓
California Verbal Learning Test-II, Short form	✓		✓	✓
Rey-Osterrieth Complex Figure–Copy Trial	✓		✓	✓
D-KEFS Color Word Test Conditions 1–3	✓		✓	✓
Exploratory and screening measures:				
To be administered by examiner:				
MINI-COG	✓			
PHQ-9	✓		✓	✓
ATHF	✓			
Mini International Neuropsychiatric Interview	✓			
CIRS-G	✓			
CGI (Severity portion only)	✓			
CGI (entire scale)			✓	✓
Athens Insomnia Scale	✓		✓	✓
MoCA (London only)	✓			✓
WAIS-IV–Test of premorbid functioning	✓			
To be completed by participant:				
FES-I (London only)	✓		✓	✓
PHQ-9	✓		✓	✓
GAD-7	✓		✓	✓
GAI	✓		✓	✓
WHO DAS 2.0	✓		✓	✓
EQ-5D-5 L	✓		✓	✓
TTO	✓		✓	✓
TSES	✓		✓	✓
PCI			✓	✓
SPWB	✓		✓	✓
FFMQ	✓		✓	✓
Likert scale			✓	
Participant-reported side effects			✓	✓
Other data collected:				
Assessment of eligibility	✓			
Informed consent	✓			
rs-fMRI/MRI/sMRI (only if enrolled in MRI sub-study)	✓		✓	
Demographics (including medications)	✓			
Changes in demographics (including medications)			✓	✓
Acti-watch^a^ (Montreal only)	✓		✓	
Gait (London only)	✓		✓	✓
Blood draw (London only)	✓		✓	

^a^this measure is administered 2 weeks prior to week 0 and 2 weeks following week 12

*MRI* magnetic resonance imaging, *rs*-*fMRI* resting state functional magnetic resonance imaging, *sMRI structural  magnetic resonance imaging*, *PHQ-9* Patient Health Questionnaire 9-item, *ATHF* Antidepressant Treatment History Form, *CIRS-G* Cumulative Illness Rating Scale – Geriatric, *HRSD-17* Hamilton Rating Scale for Depression 17-item, *CGI* Clinical Global Impression, *MoCA* Montreal Cognitive Assessment, *FES-I* Falls Efficacy Scale – International, *GAD-7* Generalized Anxiety Disorder 7-item, *GAI* Geriatric Anxiety Inventory, *WHO DAS 2.0* World Health Organization Disability Assessment Schedule 2.0, *TTO* Time Trade Off, *TSES* Toronto Side Effects Scale, *PCI* Phenomenology of Consciousness Inventory, *SPWB* Ryff’s Scales of Psychological Well-Being, *FFMQ* Five Facet Mindfulness Questionnaire, *WAIS* Wechsler Adult Intelligence Scale, *D-KEFS* Delis-Kaplan Executive Function System, *MINI* Mini International Neuropsychiatric Interview

### Primary outcome

#### Hamilton Rating Scale for Depression (HRSD-17)

The primary outcome is depression scores as rated by the HRSD-17 which is a 17-item rater-administered scale that has items scored on a scale of 1–3 or 1–5. The HRSD-17 is completed by a trained rater who assesses mood, guilt, suicidal ideation, sleep disturbances, loss of appetite, weight gain/loss, hypochondriasis, sexual impairment, anxiety, insight into depressed mood, as well as physiological symptoms of depression and anxiety. The typical duration of the interview is 20–30 min. Scores on the HRSD-17 are as follows: 0–7 (minimal or not present), 8–13 (mild), 14–18 (moderate), 19–22 (severe), and 23 + (very severe).

### Secondary outcomes

#### Rey-Osterrieth Complex Figure

This neuropsychological test asks participants to copy a complex, geometric image by copying it onto a blank piece of paper [49, 50] – the “copy trial.” Participants are then asked to redraw the image from memory after a 30-min delay – the “recall trial.” Although many different scores can be pulled from this assessment, for the purposes of this study we are focusing on those associated with executive functions as assessed by the Boston qualitative scoring system for the Rey-Osterrieth Complex Figure: the “planning” score, the “fragmentation” score, and the “organization” score. The order in which the copied/recalled image is drawn composes the planning score. For example, participants who start with larger, identifiable features (i.e., “configural elements”) score more points than participants who begin their drawing with the details (i.e., “clusters”). The continuity of pen strokes on copied/recalled image drawings creates the fragmentation score. For example, is a line drawn with a single pen stroke or did the participant have to extend the line? The fragmentation and planning scores will be then combined to generate an organization score. Age-scaled norms will be used to create scaled scores for each of these outcomes [51]. Such a scoring method has demonstrated validity among depressed older adults [52].

#### California Verbal Learning Test-II (CVLT-II), Short form

The CVLT-II is a tool used to assess verbal learning and memory that has a built-in measure of semantic organization (clustering). Participants are read to and asked to repeat a list of nine words four times aloud. Following a brief distraction task (counting backwards from 100 by 1’s for 30 s), participants will be asked to recall the list with no cue. After a 10-min delay, participants will be once again asked to recall the list of nine words. They will then be asked to recall which items in the list are fruits, then clothing, then tools. Additionally, participants will read a separate list of words that contains items from the original nine items and items that were not. Participants may answer “yes” if the word is from the original nine and “no” if it is not. Following another 5-min break, participants will read nine pairs of words and will have to identify which of the two words was on the original list of nine words. Scores will be determined by recording correct and incorrect responses on each task as well as semantic clustering during free-recall trials. Raw scores will be used to assess overall performance on the CVLT-II, with higher correct responses and lower incorrect responses indicating good acquisition and retention of verbally encoded information and higher semantic clustering scores indicating greater use of inherent semantic organization within the word list as an indicator of executive functioning. Age- and education-level-scaled norms will be used to create scaled scores for each of these outcomes [53].

#### Wechsler Adult Intelligence Scale, fourth edition (WAIS-IV)-Digit Span

The WAIS-IV-Digit Span is an attention span task in which series of single-digit numbers are read out to participants [54]. In the second task the participant will be instructed to recite a series of numbers in backward order. For the final task, the rater will ask participants to re-order the numbers in mind, from lowest to highest. Participants will score points for accurately reciting number series. Two lists of equal length (e.g., three numbers) will compose a “trial.” Participants will always attempt both number series within a trial but need only one correct response to proceed to the next trial. Correctly reciting one list is 1 point, with a total of 2 points per trial. Each task has as many as eight trials. The task is ended when participants fail a trial (e.g., two incorrect responses) and the rater then moves onto the next task. Scaled scores are generated for the total score achieved on each task (that is, forward, backward, sequencing) and the longest digit span (LDS) for each task (e.g., how many numbers are in the longest series they correctly responded to). We are also are generating a reliable digit span (RDS) score [55], which uses the number of digits read in the last trial for which they received a perfect score (both lists correctly recited: e.g., if there were four numbers in the last perfect trial, their RDS = 4). Total scores range from 0 to 48.

#### Delis-Kaplan Executive Function System (D-KEFS) Verbal Fluency Test

The Verbal Fluency Test of the D-KEFS asks participants to provide responses according to rules set out by three subtests [56]. The first subtest, letter fluency, asks participants to name as many words as possible that begin with a certain letter in 60 s. Participants perform this task three separate times for three separate letters: “F,” “A,” and “S” (names, numbers, place names, and variations of the same word are not accepted as correct responses). The category fluency subtest asks participants to list as many items they can think of within a category in 60 s. Participants perform this task twice for two separate categories: “animals” and “boys’ names.” The final subtest is the category-switching task in which participants are asked to switch between naming fruits and naming furniture. Participants, once again, have 60 s to complete this task. Participants are given points for correctly provided responses and lose points for repetitions or set-loss errors (i.e., not adhering to the rules of the subtest). In the category-switching subtest participants are additionally scored on every successful “switch” they make from fruits to furniture. A scaled contrast score will be generated which compares letter fluency (total correct) and category fluency (total correct).

#### D-KEFS Color-Word Interference Test

Three tasks from the Color-Word Interference Test of the D-KEFS will be administered to participants [56]. The first test will ask participants to name color patches (red, green, or blue; “color-naming” task). The second will ask participants to read color names on a sheet (“red,” “green,” or “blue,” “word-reading” task). The third task asks participants to read a sheet with words on it printed in colored ink (“red,” “green,” or “blue”; “inhibition” task), and instructs participants to only name the ink color in which the words are printed (red, green, or blue) and not read the word; the ink color differs from the color names. Three raw scores are taken from each task: time to complete the task, incorrect responses, and corrected responses (e.g., if a participant says the wrong color or word but immediately corrects). Scaled scores will be generated for the completion times of the color-naming, word-reading, and inhibition tasks. Scaled scores for cumulative errors made by participants during the inhibition task will also be generated. We will be using scores generated from the inhibition task in our analysis.

### Exploratory outcomes

#### Cumulative Illness Rating Scale-Geriatric (CIRS-G)

The CIRS-G is a standardized assessment system for evaluating all of the 14 body systems and the severity of illnesses with each [57]. Each are rated on a scale of 0–4; with 0 representing no issue to 4 representing severe problems with that system. Five scores are calculated for the scale; the total score, the total number of categories endorsed, the severity (ratio of total score/number of categories endorsed), and the number of categories at level 3 or level 4. A higher score for any of the five scores indicates greater problems or greater severity of problems with the various health systems [58].

#### Patient Health Questionnaire 9-item (PHQ-9)

The PHQ-9 is a 9-item, self-rated measure of depression. Total scores indicate various levels of depression: 0–4, no depression; 5–9, mild depression; 10–14, moderate depression; 15–19, moderately severe depression; 20–27, severe depression [59, 60].

#### Generalized Anxiety Disorder 7-item (GAD-7) scale

A 7-item (each scored 0–3) self-rated measure of anxiety, shown to be valid for use in the elderly. Scores range from 0 to 21. Higher scores indicate greater anxiety symptoms (5–9, mild anxiety; 10–14, moderate anxiety; 15–21 severe anxiety) [61].

#### Clinical Global Impression (CGI) scale

The CGI is a 3-item scale, psychiatric tool well validated to provide an overall assessment by a clinician of a participant’s rating of illness severity (1 = normal to 7 = among the most severely ill patients), improvement/change (1 = very much improved to 7 = very much worse), and therapeutic response (0 = marked improvement with no side effects to 4 = unchanged or worse and side effects outweigh the therapeutic effects). No global score is generated [62].

#### Athens Insomnia Scale

The Athens Insomnia Scale is an 8-item, self-rated measure of the extent of sleep difficulties with each item representing a different aspect of sleep (e.g., “Overall Quality of Sleep”). Ratings are from 0 to 4, with 0 being no difficulty with the item and 4 being the most difficulty with the item. Higher overall scores on this scale indicate higher difficulties with sleep [63].

#### Acti-watch (Montreal site only)

Participants will be outfitted with a GENEActiv Original wrist-actigraphy device by Activinsights, Kimbolton, Cambridgeshire, UK. Participants will wear the device in the 2 weeks preceding the beginning of the study intervention and for the 2 weeks following the end of the study intervention. At the end of these two time periods, we will evaluate anthropometric measures (body mass index, waist circumference). Using the GENEActiv Original software, a detailed hourly chart will be generated for each 2-week period reporting on sleep data for participants. This device will be in use at the Montreal (QC, Canada) site only.

#### World Health Organization Disability Assessment Schedule 2.0 (WHODAS 2.0)

The WHODAS 2.0 is a 36-item, self-rated questionnaire assessing a participant’s difficulties, due to physical- or mental-health issues, in various activities (e.g., “self-care”) over the past 30 days. Participants provide responses from 1 to 5, with 1 being “none” and 5 being “extreme or cannot do.” Item raw scores, raw domain scores, and domain average scores (out of 5) are generated as well as a “General Disability Score (Total)” and a global average score (out of 5). Higher scores indicate greater difficulty with the item, the domain, overall, or on average [64].

#### EuroQol-5 Dimensions-5 Levels (EQ-5D-5 L)

The EQ-5D-5 L is a two-page, self-rated assessment of a participant’s self-rated health state on that day. The first page asks participants to indicate how much difficulty they have in a health dimension (e.g., “mobility”) using one of five levels: no problems, slight problems, moderate problems, severe problems, or extreme problems. Higher scores indicate greater severity of problems with a health dimension or overall. The second page asks participants to rate their current health state on a scale from 0 (“the worst health you can imagine”) to 100 (“the best health you can imagine”). This section serves as a subjective measure of a participant’s own health [65].

#### Time Trade Off (TTO)

The TTO consists of one, self-rated question which asks participants to imagine that they have 10 years left to live. They may choose to live for 10 more years in their current state of health or give up some life years to live for a shorter period in perfect health. Participants will mark on a number line from 0 to 10 the number of years in full health that they feel is equivalent to their current state of health [66].

#### Toronto Side Effects Scale (TSES)

This 32-item scale asks about a broad variety of participant-reported side effects. The scale uses a 5-point Likert scale to assesses both the frequency (1 = “Not at all” to 5 = “Everyday”) and severity (1 = “No trouble” to 5 = “Extreme trouble”) of side effects. Frequency and severity scores for each item are then multiplied together to create an “‘intensity” score. Higher overall scores on the TSES indicate greater intensity of side effects, but do not indicate which side effects compose the intensity [67].

#### Montreal Cognitive Assessment (MoCA; London only)

The MoCA is a brief, 30-item measure of different cognitive abilities (e.g., visuospatial ability, short-term memory). The first task is a sequencing task which will ask participants to continue drawing a line following a number-to-letter pattern (e.g., 1 to A, 2 to B, etc.; the “trail-making” task). The second task will ask participants to copy a cube. The third will ask participants to draw a clock and to set the hands to 10 min past 11. The third task is an animal-naming task, in which participants will have to correctly identify depictions of a lion, rhinoceros, and camel. The remaining items include a variety of tasks: immediate and delayed word recall, attentional tasks, language tasks, abstraction tasks, and an orientation task. Total scores are generated by adding up scores from every task on the MoCA and range from 0 to 30, with scores above 26 indicating normal cognitive functioning [68].

#### Ryff’s Scales of Psychological Well-Being (SPWB)

The SPWB is a self-report measure with 54 short questions that creates scores for six dimensions of psychological well-being: self-acceptance, the establishment of quality ties to others, a sense of autonomy, the pursuit of meaningful goals and a sense of purpose in life, and continued growth and development as a person. This measure is scored on a 6-point scale from “Strongly disagree” to “Strongly agree.” These scales are regarded as separate dimensions of psychological well-being but can also be aggregated for a global score [69].

#### Five Facet Mindfulness Questionnaire (FFMQ)

The FFMQ is a 29-item, self-rated questionnaire that examines five factors of mindfulness including observe, describe, act with awareness, non-judgmentality, and non-reactivity. Each question is scored from 1 (never or rarely true) to 5 (very often or always true). Total scores range from 29 to 145, with higher scores indicating greater overall mindfulness and higher scores within a factor indicating higher levels of that factor, in particular (e.g., the observe factor) [70].

#### Phenomenology of Consciousness Inventory (PCI)

The PCI is a self-report measure containing 53 items about an immediately preceding subjective experience, scored on 7-point scales between opposing statements. The items are organized into 21 scales that include altered states of awareness, self-awareness, altered experiences, volitional control, rationality, internal dialog, positive affect (which includes joy, love, and sexual excitement), negative affect (which includes anger, sadness, and fear), imagery, attention, memory, and arousal. Scores on the scales will be used to assess the degree to which a person has experienced an altered state of consciousness [71].

#### Blood draw

Two vials, approximately 10 ml, of whole blood will be taken from participants by individuals trained in handling and extracting biological samples. Samples will be labelled with a de-identified participant ID, packaged, and shipped to a secure blood bank located at Robarts Research Institute (RRI) in London, ON, to be analyzed at a later date. Samples will be centrifuged at RRI for plasma extraction within 6 days of collection and stored at − 85 °C. The samples will be sent via secure delivery services in London, ON, to ensure safety of samples and participant IDs. An enzyme-linked immunosorbent assay (ELISA) kit will be used to measure any number of blood-borne inflammatory markers. As such, levels of C-reactive protein, interleukin (IL)-10, IL-1β, IL-6, tumor necrosis factor alpha (TNF-α), and epidermal growth factor-1 (EGF-1), may be measured. Indicators of the hypothalamus-pituitary-adrenal (HPA) axis activation and stress, such as cortisol, dehydroepiandrosterone, adrenaline, noradrenaline, and aldosterone levels, may also be tested.

#### Brain scanning

A 7-Tesla MRI machine, located at RRI in London, ON, will be used to gather magnetic resonance spectroscopy (MRS), functional, and structural data of the hippocampi and the posterior cingulate cortices (PCCs), as well as fronto-striatal connectivity at the resting state. During the scanning session, instructions will be presented to the participant through MRI-compatible headphones. The scanning session will include: a three-dimensional Magnetization Prepared Rapid Acquisition Gradient Echo (MPRAGE) scan for structural measurements; 8 min of a T2 (echo planar imaging (EPI))-sequence during the eyes-open resting state; and a MRS from the hippocampal and PCC voxels collecting using simplified localization by adiabatic selecting refocusing (semi-LASER), in line with a previously established MRS protocol [72]. MRS will be used to measure levels of glutathione (GSH). Given the time for equipment set-up and scanning, scanning procedures are expected to take close to 1 h for completion.

#### Magnetic Resonance Environment Screening Form

This is a Western University Centre for Functional and Metabolic Mapping questionnaire that participants need to complete if they wish to participate in the MRI portion of this study. It surveys surgical history as well as the presence of medical implants that may be impacted by MRI scanning (e.g., pacemakers) or otherwise problematic implants (e.g., metal implants).

#### Falls Efficacy Scale – International (FES-I)

The FES-I is a 16-item, self-rated scale used to assess fear of falling. Participants are asked about their fear of falling while doing various daily tasks (e.g., “Going up or down stairs”) and rate items on a scale of 1 (“Not concerned at all”) to 4 (“Very concerned”). Scores range from 16 to 64, with higher scores indicating greater fear of falling [73].

#### Gait

Outcomes include mean stride length and mean gait velocity. Both stride length and gait speed will be measured using a GAITRite® portable walkway (CIR Systems Inc., Franklin, NJ, USA). This device utilizes embedded pressure sensors that electronically collect necessary information. Participants will be asked to walk along the walkway and perform various tasks in a single length of the device: (1) complete a normal walk down the walkway; (2) count backwards from 100 by 1; (3) name as many animals as possible; (4) count backwards from 100 by 7; and (5) walk as fast as possible without running down the walkway. A normal walking pace will be gathered using the device prior to the above tasks. This task will be completed at the London (ON, “Canada”) site only and will be conducted in the Brain and Gait laboratory of Dr. Manuel Montero-Odasso, located at the Parkwood Institute in London, ON, Canada.

### Medication adherence

Participants will be asked to remain on their antidepressant (or other prescribed) medications for the duration of the study. Adherence to this will be determined at each follow-up as participants will be asked if there have been any changes to medication. Information on the medication, dose, frequency, reason, start, and end date of all medications (over-the-counter, prescribed) will be collected.

### Randomization and blinding

After baseline assessments, participants will be randomly assigned to the SSM or HEP arm of the study. Group allocation will be stratified by site (London or Montreal). Additionally, participants will be stratified to the presence of treatment resistance (TR) or not. Stratification and randomization will be conducted by an independent statistician based in London, ON. An independent member from administrative staff at the London site will receive the list of randomization and save it on a secure electronic database with access available to the principal investigator (PI) only if the code needs to be broken in an emergency. This process will ensure that group allocation remains concealed from raters, clinicians and care providers. Due to the nature of the intervention being behavioral in nature, it is not possible to blind participants to study arm assignment.

Participants will be informed by telephone of group allocation by an administrative assistant not involved in the study. Alternatively, participants will be handed a sealed envelope (prepared by the same administrative assistant) by a research assistant.

## Interventions

The interventions will begin after 4–10 eligible participants (per intervention; 8–20 total) have been recruited into the study and have attended a baseline assessment session. Both interventions will require the attendance of 2-h training sessions over four consecutive days. Participants will also attend 1-h follow-up sessions once per week for the remaining 11 weeks and be asked to practice the trained technique at home for 40 min per day. “Practice logs” will be provided to participants to record their daily practice of the trained technique.

### *Sahaj Samadhi* meditation (SSM) – intervention group

This is the experimental arm of the study. It will be delivered at both sites by trained, certified non-clinician instructors available from our collaborative partner, The Art of Living Foundation. During the first day of training, participants will learn the nature of meditation and then have a personal, guided meditation using a mantra, to be brought into their awareness, if the need arises. On days 2–4, there will be a focus on understanding the nature of the mind and the thoughts arising from it, additional guided meditation by the instructor(s), and a discussion of correct/incorrect meditation practices. The first 4 days of training will be 2 h each. Weekly follow-up sessions (1 h) will include 20 min of guided meditation practice. Participants will be trained and be able to discuss with instructors how to respond to the experiences that arise during meditation, what enhances or detracts from effective meditation, and review methods to support meditation at home. The uniqueness of SSM arises from the ultimate goal of quieting the mind until an individual is “aware of awareness.” The goal of SSM is to attain a state of automatic self-transcendence. All SSM participants will be asked to practice SSM at their own homes, twice a day, for the duration of the study. Compliance with this request will be reviewed through assessment of daily practice logs given to all participants.

### Health Enhancement Program (HEP) – active control

The HEP has been designed as a manualized, active control in this study as well as other meditation-based intervention trials. This intervention will allow us to control for several non-specific factors found in a meditation group intervention such as group support/morale, behavioral activation, reduction of stigma, facilitator attention, treatment duration, and time spent on at-home practice. Additionally, we will ensure that HEP matches the time commitment of SSM. The HEP modules will include information sessions on health promotion, healthy diet, self-care, music, and exercise. However, participants will not learn any breathing techniques or meditation. All HEP participants will also be asked to complete daily practice logs. Time commitments to the HEP are identical to those for SSM: 4 days of 2-h training sessions followed by 1-h follow-up sessions for 11 weeks, coupled with 40 min of daily at-home practice.

### Participant adherence to intervention

Daily practice logs will be provided to participants for them to fill out in their own time. Participants will log the days in which they practiced SSM/HEP techniques.

## Statistical analysis

### Sample size

Recruitment is expected to occur over a 26- to 36-month period at a rate of two participants per week (total of *n* = 192). See Fig. 1 for a schematic diagram. It is estimated that a sample of 192 participants will offer sufficient power for the primary outcome (HRSD-17). This sample size calculation is based on an estimated effect size from our pilot data from participants with LLD undergoing SSM or treatment as usual (TAU). Over the 12-week intervention, participants in the SSM group had significant change in HRSD-17 scores when compared to the TAU group (2.66 (95% CI 0.26 to 5.05) point greater reduction; Cohen’s D = 0.61, *p* = .030). Assuming an average cluster size of 4–10 participants and a 1:1 allocation between the SSM and HEP groups, 192 participants will allow us to observe a 2.6-point difference in HRSD-17 scores between the two groups at a two-tailed α = 0.05 and 1 − β = 0.80. A 2.5-point difference is considered to be clinically significant among consensus expert reports and the Food and Drug Administration (FDA) considers it a minimum standard [74]. A 16% rate of attrition for this study is a conservative estimate based on our previously observed 13% attrition rate in the SSM versus the TAU study. As such, it is predicted that approximately 160 of the planned 192 recruited participants will complete the study and we will still have adequate statistical power.

### Primary outcomes

The HRSD-17 scores at baseline, 12-week, and 26-week follow-up of participants assigned to SSM and HEP will be compared using linear mixed models: 2 (SSM vs HEP) × 3 (0 weeks, 12 weeks, 26 weeks). This approach will account for the clustering of observations, since both SSM and HEP will be delivered in a group setting. Next, we will evaluate whether a greater proportion of participants assigned to SSM, compared to HEP, achieve depression remission (defined as a HRSD-17 score ≤ 7 at the 12-week follow-up) using generalized linear mixed models (hypothesis 1B).

### Secondary outcomes

Each measure of executive functioning will produce a Z-score from collected data. Z-scores will be compared using linear mixed models: 2 (SSM vs HEP) × 3 (0 weeks, 12 weeks, and 26 weeks). Z-scores for each of the  executive functioning measure will be combined; the mean of the summated Z-scores will then be used to generate an overall Z-score for executive function.

### Fidelity/confidentiality

Data will be collected and stored using Research Electronic Data Capture (REDCap), a secure, online data collection tool (access provided by Lawson Health Research Institute in London, ON). Raters will also use REDCap to administer assessments and capture participant data. For assessments that are required to be completed on paper forms (i.e., executive function measures), pertinent data (i.e., Z-scores) will be inputted by the research personnel into REDCap. For assessments that are self-rated, participants will complete a REDCap-generated survey. Collected data will be continually reviewed by the research staff to ensure accuracy of completion. All changes in REDCap will be automatically logged to ensure the fidelity and integrity of the study data. As REDCap is a secure, online data-entry and repository tool, it will be available for access in real time by researchers in both London, ON, and Montreal, QC.

### Participant retention efforts

Exit interviews will be conducted at week 12 for all withdrawn participants willing to attend. These exit interviews will include all week 12 study procedures. Study participants will be remunerated at a rate of CAD$20 per assessment visit for the main study and CAD$80 per visit if participating in the MRI sub-study. To prevent undue burden, participants will be offered regular breaks during study assessment procedures as well as flexibility of attending these procedures either over one whole day or two consecutive half days, based on their personal preference. It is expected that the baseline assessment procedure will need 6–8 h of participant time while follow-up assessments at 12 and 26 weeks will each be 4–6 h long. If a participant chooses to withdraw at any time during the study, they will be allowed to do so. However, the research staff will try and ascertain from the participant the reason for withdrawal and document these reasons. If a participant is unable to adhere completely with this study protocol, all reasonable efforts will be made to accommodate their needs and requests. If there is significant deviation (greater than 1 week from an expected assessment point), they will be considered as a withdrawal from the study. All such withdrawals and deviations will be recorded on REDCap.

### Training of research staff

Assessors will be trained by the PIs (AV and SR) and appropriate co-investigators at their respective sites on the delivery, administration, and recording of all study procedures and scales. Convergence of ratings will be tested via intraclass correlation coefficients (ICCs) for all raters at study commencement. Calculation of ICCs will be ongoing to ensure the fidelity and reliability of study results throughout the duration of the study.

### Risk screening

Participants are asked about suicidal ideation at the very first appointment. Suicidal ideation, gestures, or any other behavior/thoughts/disclosures that may elevate the risk of suicide will be addressed by contacting the site-appropriate PI – both are trained psychiatrists – to determine participant risk. Participants are also screened for substance and alcohol abuse. Qualifying for either renders a participant ineligible as they would likely not be able to meaningfully participate in the intervention and may pose a risk to themselves or other participants should they be so dependent that they attend study intervention sessions under the influence of substances or alcohol. Any untoward adverse effects or deterioration in mental health posing as an imminent risk (e.g., elevated risk of suicide) – will be promptly brought to the attention of the site PIs for further assessment and/or intervention.

### Focus groups

Participants will be invited to attend a focus group at the end of each study primary outcome assessment point (12 weeks). To evaluate the information gathered from the focus group at the end of the intervention, a thematic analysis of the transcripts from the focus group will be conducted. Transcribed responses will be divided into “recording units,” which will consist of groupings of words or sentences corresponding to one category. Five predetermined content categories will be analyzed using a content-analysis grid containing five content categories, including: intervention/program environment, clinicians, intervention, program organization, and general comments about the program. Attained information from these transcripts will serve to improve interventions, study processes, and further reduce any possible risk to participants.

## Discussion

The current protocol describes a newly funded research project which will assess the efficacy of SSM compared to HEP in reducing depressive symptoms, improving neuropsychological symptoms as well as inform key neurobiological mechanisms of action. This study builds on our previous RCT of SSM compared to TAU which found that SSM had three times higher remission rates (40.0% vs 16.3%; odds ratio, 3.36; 95% CI 1.06–10.64; *p* = 0.040), as an augmentation strategy compared to TAU alone, in patients with LLD. Additionally, SSM was found to be easy to learn, can be taught to a group, and is scalable. As such, SSM can, therefore, be positioned to be offered as an augmentation therapy to TAU for individuals with LLD and TR-LLD [37, 39].

This study protocol describes the use of an active-control group, rater blinding, and validated tools to collect depression-related data, executive function scores, as well as a number of exploratory outcome measures. Our study protocol should allow us to collect this data reliably and with a high degree of fidelity. This protocol offers a brief outline of the planned study procedures. We hope to not only assess the effects of SSM through a rigorously designed RCT including an active-control group, but also to collect crucial initial data (e.g., neuroimaging and serum biomarkers) regarding the mechanisms of action of this intervention. If we find positive effects of SSM, it will offer a better understanding of the extent of response with SSM and/or an active-control intervention, HEP.

Our extensive battery of assessments will allow us to measure neuropsychological changes associated with the effects of SSM and/or HEP. In addition, we will be able to assess the effects on other key pathophysiological variables, including the planning and organization components of executive function, gait, sleep, mindfulness, consciousness, structural, functional, and MRS imaging (details available on request), as well as measurement of anti-inflammatory markers.

Our battery of neuropsychological tests and neuroimaging of brain regions associated with executive function [33] may aid future studies in better understanding the potential benefits of SSM and/or HEP on these processes and systems. Our selection of key executive function tasks is inclusive and gives adequate priority to the assessment of planning and organization, key cognitive domains affected in LLD and TR-LLD [20]. Our study protocol will also allow us to correlate changes, if any, in GSH, with simultaneous changes in brain volume and functional imaging in the hippocampi and the PCCs. To our knowledge, this is the first time that a comprehensive neuropsychological and neuroimaging battery is being offered in patients with LLD and/or TR-LLD receiving a meditation-based intervention. Findings from this study could hence potentially lead to a better understanding of the link between neuroinflammation and brain changes associated with LLD.

By examining other physiological outcomes, such as gait, consciousness, sleep, and blood inflammatory markers, we will be building on our understanding of how LLD affects a number of key central and peripheral organ functions.

Overall, if the planned study is successful, there would be evidence to support SSM as an augmentation to TAU among older adults with LLD and/or TR-LLD. The other main benefits of this study would be to offer valuable insight into the potential applicability as well as mechanism of action of SSM in routine clinical practice. Future studies could examine the cost-effectiveness of this intervention.

## Conclusion

Our trial could help in definitively demonstrating the efficacy of SSM in improving symptoms of LLD when compared to an active-control group that accounts for receiving care and social support in a group setting. Additionally, our neuropsychological, neurobiological, and physiological outcomes could help inform our understandings of the mechanisms and comorbidities of LLD, as well as its biological basis.

## Trial status

This protocol manuscript is based on an internal protocol document: “Alternative Treatments to Help Late-Life Depression Study Protocol” (version 2; 13 June 2018). This document is the first version, last edited on 30 January 2019. Recruitment began in late June 2018 and is expected to be completed in June of 2020.

## Trial progress

As of 22 July 2019, 44 participants have been enrolled in the study. Twenty-six participants have completed the study, seven participants are currently undergoing the study intervention, and 11 participants have withdrawn from the study.

## Data Availability

Not applicable.

## Abbreviations

ASTM: Automatic self-transcendental meditation
ATHF: Antidepressant Treatment History Form
CGI: Clinical Global Impression
CIRS-G: Cumulative Illness Rating Scale-Geriatric
CVLT-II: California Verbal Learning Test-II
D-KEFS: Delis-Kaplan Executive Function System
EGF-1: Epidermal growth factor-1
ELISA: Enzyme-linked immunosorbent assay
EPI: Echo planar image
EQ-5D-5 L: EuroQol-5 Dimensions-5 Levels
FES-I: Falls Efficacy Scale – International
FFMQ: Five Facet Mindfulness Questionnaire
GAD-7: Generalized Anxiety Disorder 7-item
GSH: Glutathione
HEP: Health Enhancement Program
HPA: Hypothalamus-pituitary-adrenal
HRSD-17: Hamilton Rating Scale for Depression
ICC: Intraclass correlation coefficient
IL: Interleukin
LDS: Longest digit span
LLD: Late-life depression
LOI: Letter of information
MINI: Mini International Neuropsychiatric Interview
MoCA: Montreal Cognitive Assessment
MPRAGE: Magnetization Prepared Rapid Acquisition Gradient Echo
MRI: Magnetic resonance imaging
MRS: Magnetic resonance spectroscopy
PCC: Posterior cingulate cortex
PCI: Phenomenology of Consciousness Inventory
PHQ-9: Patient Health Questionnaire 9-item
RCT: Randomized controlled trial
RDS: Reliable digit span
REDCap: Research Electronic Data Capture
RRI: Robarts Research Institute
SPWB: Ryff’s Scales of Psychological Well-Being
SSM: *Sahaj Samadhi* meditation
SSRI: Serotonin-reuptake inhibitors
TAU: Treatment as usual
TNF-α: Tumor necrosis factor alpha
TR: Treatment resistance
TR-LLD: Treatment-resistant late-life depression
TSES: Toronto Side Effects Scale
TTO: Time Trade Off
WAIS-IV: Wechsler Adult Intelligence Scale, fourth edition
WHODAS 2.0: World Health Organization Disability Assessment Schedule 2.0
